# Editorial for the special collection: frontiers in rare disease genetics

**DOI:** 10.1186/s44342-025-00039-2

**Published:** 2025-03-07

**Authors:** Murim Choi

**Affiliations:** Department of Biomedical Sciences, Seoul National University College of Medicine, Seoul, 03080 Republic of Korea

Rare diseases collectively affect millions of individuals worldwide, presenting significant challenges to both medical research and clinical practice. Despite their rarity on an individual level, the aggregate impact of these conditions underscores the urgent need for innovative solutions in diagnosis, treatment, and management. The articles featured in this issue spotlight remarkable advancements in genomics and precision medicine, illustrating how cutting-edge technologies and collaborative efforts are transforming the landscape of rare disease research.

Jungmin Choi’s review highlights the critical role of genomic technologies in understanding rare diseases [1]. The integration of big data analytics and advancements in artificial intelligence and machine learning has propelled diagnostics to new heights. Large consortium initiatives have not only expanded our knowledge-base but also fostered collaborative frameworks essential for tackling challenges such as data sharing and privacy. The emphasis on secure data practices reflects the delicate balance between innovation and ethical responsibility. As genomic research continues to evolve, it paves the way for precision medicine approaches that tailor therapies to individual patients.

Jin Sook Lee provides a clinician’s viewpoint on the molecular diagnostic approach to rare neurological diseases [2]. The article underscores the importance of phenotyping alongside genomic testing, emphasizing that traditional clinical expertise remains invaluable even in the genomics era. Long-read sequencing technologies, particularly beneficial in diagnosing repeat expansion disorders and complex structural variants, have emerged as powerful tools. Additionally, the necessity for periodic reanalysis and the integration of genomics with multi-omics studies underscores the dynamic nature of molecular diagnostics. These approaches hold promise for addressing undiagnosed cases, ultimately enhancing patient care.

Anna Cho’s article sheds light on the advancements in genomics-driven research for neuromuscular diseases (NMDs) [3]. The dramatic increase in the identification of genetic causes—from fewer than 30 in the 1990s to over 600 today—illustrates the transformative impact of next-generation sequencing technologies. The advent of gene therapies, such as those for spinal muscular atrophy and Duchenne muscular dystrophy, marks a significant milestone in the treatment of NMDs. Despite these successes, challenges such as genetic heterogeneity and the high costs of therapies remain. Continued innovation in gene and RNA-based therapeutics is essential for overcoming these hurdles and improving patient outcomes.

Finally, Heon Yung Gee’s exploration of the genetic etiologies of sensorineural hearing loss in Koreans offers valuable insights into the genomic complexities of this common sensory disorder [4]. The article reviews the clinical characteristics and genotype-phenotype correlations, emphasizing the role of allelic heterogeneity and variable expressivity in diagnosis. Moreover, genome-wide association studies have identified potential genetic factors linked to age-related hearing loss, paving the way for targeted therapeutic interventions. These findings highlight the importance of understanding population-specific genetic variations in advancing precision medicine.

The review articles presented in this issue underscore the transformative potential of genomics and precision medicine in addressing the challenges posed by rare diseases. They also function as the bridging articles of the rare disease endevor that our journal has consistently explored [5–9]. However, the journey is far from over. Overcoming barriers such as data privacy concerns, high costs, and genetic heterogeneity requires sustained collaboration across disciplines and regions. By fostering partnerships and leveraging emerging technologies, the scientific and medical communities can continue to make strides toward improving the lives of individuals affected by rare diseases.
